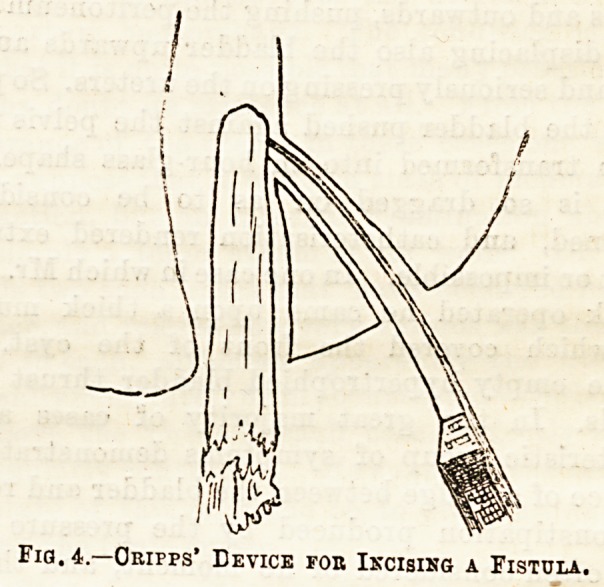# Ischio-Rectal Abscess and Fistula

**Published:** 1895-11-23

**Authors:** E. Percy Paton


### MEDICAL PROGRESS AND HOSPITAL CLINICS.

[*The Editor will be glad to receive offers of co-operation and contributions from members of the profession. All letters should be addressed to* THE EDITOR, AT THE OFFICE, 428, STRAND, LONDON, W.C.]

### ISCHIO-RECTAL ABSCESS AND FISTULA.

By E. PERCY PATON, M.D., M.S., F.R.C.S.

(*Continued from page* 116.)

The examination of a patient with fistula requires some little care in order to make out the exact condition of affairs which is present. It is always best in the first place to have the patient lying down instead of examining him standing up, as is so often done. Then the probe should be passed into the fistula very gently, and allowed as far as possible to find its own way, as a false tract is very easily made and will prevent the real one and its opening into the bowel, if present, from being discovered. Again, in trying to get the probe through the internal opening the instrument should always be passed before the finger is inserted into the rectum, so that the parts may be as far as possible in a quiet normal condition without any active contraction of the sphincters, as this contraction often renders the internal opening more difficult to find if it be present. As in a blind internal fistula there is no external opening another method of investigation is needed. In these cases in addition to the symptoms mentioned above of escape of pus from the anus there is always some induration or brawniness to be felt over the affected ischio-rectal fossa. When this is felt the internal opening must be sought for by bending a silver probe into a hooked form, and then by careful trial hook it into the internal opening, the side of the rectum on which the induration is being carefully and systematically explored and great gentleness being used.

**Treatment.**—Few of these fistulæ when once established will close up without operative interference, but as some patients will not permit this form of treatment the lines on which an attempt may be made to close them without will here just be alluded to. One of the most important things is to have and maintain a free external opening. The opening for this purpose may be dilated with sinus forceps; the sinus may then be irritated by inserting a probe which has been coated with silver nitrate, or injecting strong carbolic acid, and then the opening kept widely patent by a piece of pewter wire bent into a U shape, with the vertical limbs of the U close together, the other portion forms a kind of flang which prevents the plug from slipping right to the sinus. Allingham advises a plug of bone, shaped like a large collar stud and bored through the centre. This, he says, becomes self-retaining after it has been in a short time. Another method, without the use of the knife, is by passing a piece of rubber cord through the fistula into the gut, and then out of anus, and knotting it firmly, so that by its pressure it will cut its way out, and so do what could have been done by one stroke with the knife.

All the above methods, however, are very uncertain in their results and not to be depended upon, and we must now describe the more satisfactory operative treatment. The question which here first meets us is, Should all cases be treated by operation? The answer to this question must be, No. For in a certain number of patients who have advanced constitutional troubles, such as phthisis, the result of operation will not be satisfactory, either to the local or general condition, at any rate, in many cases; and this being so, it is better to use less active, or merely palliative, measures. In all other cases, however, active treatment is called for, and the principles on which this treatment is undertaken are, by laying the sinus freely open to ensure its closure from the bottom, and also the ability to keep it clean, while at the same time rest is obtained by division of the sphincter.

The details of the treatment are as follows: The bowels are well cleared before the operation by castor oil the night before, and an enema in the morning. The patient is then put in lithotomy position, the fistula, if of the blind kind, is made complete, if external by forcing a probe pointed director through into the rectum; if internal, by hooking a bent probe into the internal opening as described above, and then cutting down on its point from outside. A probe-

FIG. 4.—CRIPPS' DEVICE FOR INCISING A FISTULA.

pointed director is then passed from the ischio-rectal fossa through the sinus into the rectum and out at the anus, and then all the intervening tissues are divided by passing a bistoury along the groove of the director. In some cases it is very difficult owing to the internal opening being high up, to get the point of the director out at the anus, in which case the following device described by Cripps may be used: A piece of ordinary fire-wood is rounded and smoothed at its extremity and passed carefully into the rectum, a sharp-pointed bistoury is then passed along the sinus and made to penetrate the wood (see Fig. 4), and then both wood and knife are forcibly removed together, thus dividing the intervening tissues. A careful investigation must now be made to see if there are any secondary channels, which, if found, must be slit up. This is especially the case with any sinus which may run up beside the gut above the internal opening of the primary fistula. In searching with a probe for these secondary sinuses no force must be used, as it is very easy to make artificial sinuses, which cannot be distinguished from the real article. During the above procedure the bleeding is often very free, but as every part of the wound is well in sight, it can easily be controlled either by pressure or by the use of pressure forceps. In old-established sinuses with hard cartilaginous walls it is advisable not only to slit them up but also either to scrape out the indolent tissue with a Volkmann's spoon, or to divide the base. The dressings, consisting of well-disposed strips of gauze, should now be applied, while in the centre passing into the bowel, a good-sized piece of drainage tube is placed with a string attached to prevent its getting lost in the rectum. This serves the double purpose of allowing any bleeding which takes place to appear externally at once and at the same time permitting the patient to pass flatus with comfort. Over all is then put a pad and T bandage. The bowels are now kept confined for three or four days, the first packing, the quantity of which has been reduced after the first twenty-four hours, having been removed the day before the bowels are open. Subsequently the wound is kept lightly packed, not stuffed, with a little gauze or wool, this being applied in such a manner as just to prevent the edges coming together and to compel healing from the bottom. If at the later stages the wound becomes indolent it may be stimulated by nitrate of silver or other more or less caustic applications. The patient should be confined to bed or the sofa for at least a week, and in bad cases for considerably longer.

## Figures and Tables

**Fig. 4. f1:**